# Microfluidic SlipChip device for multistep multiplexed biochemistry on a nanoliter scale†

**DOI:** 10.1039/c9lc00541b

**Published:** 2019-08-23

**Authors:** Dmitriy V. Zhukov, Eugenia M. Khorosheva, Tahmineh Khazaei, Wenbin Du, David A. Selck, Alexander A. Shishkin, Rustem F. Ismagilov

**Affiliations:** a Division of Chemistry and Chemical Engineering, California Institute of Technology, 1200 E. California Blvd, Pasadena, CA, 91125, USA; b Division of Biology and Biological Engineering, California Institute of Technology, 1200 E. California Blvd, Pasadena, CA, 91125, USA; c Department of Chemistry and Institute for Biophysical Dynamics, The University of Chicago, 929 East 57th Street, Chicago, Illinois 60637, USA

## Abstract

We have developed a multistep microfluidic device that expands the current SlipChip capabilities by enabling multiple steps of droplet merging and multiplexing. Harnessing the interfacial energy between carrier and sample phases, this manually operated device accurately meters nanoliter volumes of reagents and transfers them into on-device reaction wells. Judiciously shaped microfeatures and surface-energy traps merge droplets in a parallel fashion. Wells can be tuned for different volumetric capacities and reagent types, including for pre-spotted reagents that allow for unique identification of original well contents even after their contents are pooled. We demonstrate the functionality of the multistep SlipChip by performing RNA transcript barcoding on-device for synthetic spiked-in standards and for biologically derived samples. This technology is a good candidate for a wide range of biological applications that require multiplexing of multistep reactions in nanoliter volumes, including single-cell analyses.

This paper explores a versatile microfluidic SlipChip device that performs multistep biochemical reactions in a multiplexed format on a nanoliter scale. Combining reagents is a basic unit operation in chemistry, yet controlled multistep merging of nanoliter droplets has remained a challenge. To move the field of microfluidics forward and increase the adoption of miniaturized platforms, we need to expand the arsenal of methods for performing this operation. The ability to perform multistep biochemical reactions will be of particular benefit for many protocols and biological assays,^2^ such as nucleic acid^3^ and biomarker detection/quantification,^4^ time-sensitive and autocatalytic^5^ reactions, as well as particle synthesis.^6^ Multistep reactions in very small volumes are also needed for low-input applications such as tissue-extracted rare-cell studies,^7^ where controlled mixing of two or more reagents is often required to start/quench reactions or to dilute solutions. Specifically, temporal control over several sequential, parallelized reactions is important in complex biochemical procedures, such as single-cell analyses.

Current solutions for merging of nanoliter droplets, such as integrated fluidic circuits and automated pipetting systems, require complex microfluidics and control systems (*e.g.* pumps, pneumatic valves, multilayer soft lithography, surface acoustic waves, microsolenoid dispensers, electrowetting-on-dielectric technology, *etc.*), especially when there are multiple steps.^6,8–14^ Although droplet-encapsulation methods^15^ provide virtually unlimited scalability to the number of compartments, and have progressed greatly in high-throughput detection,^16–18^ washing,^19^ and sorting,^20^ they have limitations with respect to reagent additions to the originally encapsulated volumes, imaging, and reaction parallelization.

SlipChip^21^ is an attractive platform on which to build multistep capabilities. In addition to offering the general, well-established benefits of a miniaturized platform, (*e.g.*, small reagent volumes, high relative concentrations of analytes),^22–24^ SlipChip devices give the user the capability to program a complex protocol of fluidic manipulations and to execute it by simply “slipping” the plates of the device among different conformations. SlipChip devices have been well characterized;^21,25–27^ briefly, a SlipChip is composed of two glass plates with microfabricated features such as wells, channels, and ducts. The volumes and shapes of these features can be tuned by photolithography. The fabricated plates are rendered hydrophobic and oleophilic with silane, and assembled with oil coating all features. Thus, when an aqueous solution is loaded into a SlipChip, it does not wet the surfaces (*i.e.*, a thin layer of oil remains between the aqueous phase and the glass surfaces). By slipping one plate with respect to the other plate (along the *x* and/or *y* axes), numerous pre-programmed configurations can be accessed and transient well-channel networks can be used to create and merge droplets. Multistep SlipChip protocols involving serial dilutions have been demonstrated.^28,30^ This work adds a capability to perform complete droplet transfer (previously droplets were split by slipping and retained in the wells) in addition to the option to retrieve samples after reaction without cross-contamination. SlipChips have also been demonstrated for multiplexing.^26,28,29^

Building on the SlipChip platform, we designed a device that accurately meters and manipulates nanoliter volumes of reagents and adds them to reaction wells in a multiplexed, parallel fashion. This multistep device facilitates complete droplet transfer between microwells by controlling the shape of the interface between two immiscible fluids^27,31^ *via* judiciously selected geometries. The parallelization of the reactions is enabled by three components: (1) surface-energy traps^32–34^ that immobilize an array of droplets while the next set of droplets is being merged with it, (2) differences in the depths (along *Z*-dimension) of the device’s microfeatures that provide the driving force for fluid transfer,^27^ and (3) well shapes (in *XY*-plane) that prevent droplet break-up and guide droplets to merge. Importantly, the device contains a set of features into which the user can pre-spot reagents that can be used to uniquely identify the contents of any one well from any other well after they are pooled. So instead of offering on-device readout only as demonstrated previously,^9,28,30^ this device is designed for the user to be able to extract the products of on-device reactions for further analysis. We demonstrate the utility of this multistep device to perform multiplexed reactions by performing a seven-step workflow: transcript barcoding for multiplexed cDNA sequencing library preparation for total RNA sequencing (RNAtag-Seq).^35^

## Results

### Sequential drop-in of reagents is driven by capillary (Laplace) pressure

**Fig. 1:**
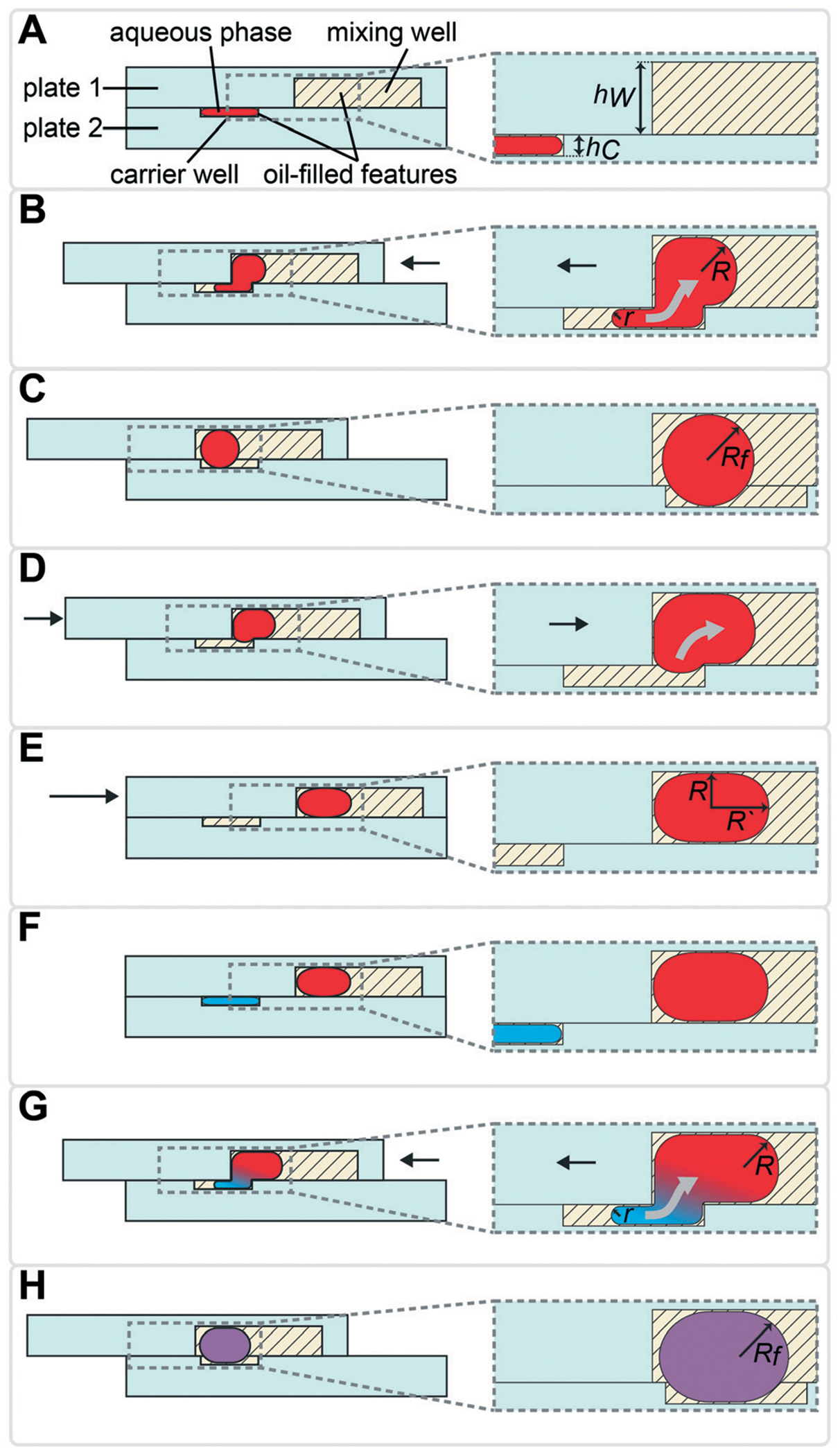
Diagram of the multistep SlipChip device illustrating the drop-in approach using back-and-forth slipping in which the interfacial energy between two immiscible phases drives fluid transfer. (A) The carrier wells are loaded with the first solution through connecting channels, which are located in a different plane (not shown). In the carrier wells, the radius of curvature of the interface in the plane shown is restricted by well height (*h*_C_). Mixing wells are deeper than carrier wells (*h*_W_ > *h*_C_) and are less restricting. (B) As plate 1 is slipped relative to plate 2, the droplet is transferred to the mixing well and is free to assume higher radius of curvature. The oil from the mixing well replaces the droplet in the carrier well. (C) Slipping is complete and the first solution is dropped-in, it is now contained in the volume created by aligned carrier and mixing wells. (D) Plate 1 is slipped back into loading position. The droplet remains in the deeper mixing well where it can assume a more energetically favorable conformation. (E) Slipping is complete and the droplet remains in the mixing well. (F) Loading carrier wells with the second solution. (G) Slipping plate 1 relative to plate 2 drops-in the second solution into mixing wells, where it merges with the droplet of the first solution. (H) The slipping is complete and both solutions are mixed and contained in the volume that aligned carrier and mixing wells create.

To sequentially add (“drop-in”) and mix multiple rounds of reagent droplets from carrier wells into mixing wells, we harnessed the driving force of capillary (Laplace) pressure by confining the interface between two immiscible fluids. One basic requirement for drop-in to work is that mixing wells must be deeper than carrier wells (Fig. 1). A droplet’s surface energy is proportional to its interfacial area, which is at a minimum when the droplet is spherical. Droplets are first loaded into shallow carrier wells (Fig. 1A) with a micropipette. These carrier wells shape the interface of the droplets and have high surface energy. Once these flattened droplets are slipped into the more spacious geometric region (mixing wells) they become more spherical (Fig. 1C), thereby attaining lower surface energy. Droplets are thus energetically incentivized to fully transfer to and remain inside the larger mixing wells as the plates are slipped back into the original conformation (Fig. 1D). Specifically, the driving force for this transfer arises from the imbalance in capillary pressures created by non-equal feature dimensions in the front and back of the droplet, as we have described previously.^27^

(1) $$\text{Δ}P_{\text{cap,back}}-\text{Δ}P_{\text{cap,front}}=2\gamma \;\cos\;\theta_{\text{R}}\left(\frac{1}{h_{\text{w}}}+\frac{1}{w}\right)-2\gamma \;\cos\;\theta_{\text{A}}\left(\frac{1}{h_{\text{C}}}+\frac{1}{w}\right)$$

$\text{Δ}P_{\text{cap,back}}$ = capillary pressure at the back of the droplet [Pa]

$\text{Δ}P_{\text{cap,front}}$ = capillary pressure at the front of the droplet [Pa]

γ = liquid–liquid interfacial tension [N m^−1^]

$\theta_{R}$ = receding contact angle [rad]

$\theta_{A}$ = advancing contact angle [rad]

$h_{\text{W}}$ = height of the mixing wells [m]

$h_{\text{C}}$ = height of the carrier wells [m]

w = width dimension $\left(h_{i}<w\right)$, assumed constant for both types of wells [m]

This drop-in approach may be performed using the same carrier well multiple times (as described in Fig. 1), or by using different carrier wells, as we describe below. Using the same carrier well is convenient when the same volume needs to be loaded for each reagent. To load different volumes of reagents, additional carrier wells of different volumes can be added to plate 2 and loaded/slipped sequentially. Note that the actual volume and the viscosities of both fluid phases are not a part of the above equation for the driving force. However, they are important factors in the viscous drag force that will be slowing the fluid transfer and should be carefully considered by the chip designer, since the resulting flowrate may become unacceptably slow for some applications. For reference, in rectangular channels $i\left(h_{i}<w\right)$ that are connected in series, the total flow resistance can be calculated by eqn (2):^27,36^

(2) $$P_{\text{drag}}/Q=\underset{i}{\sum }\frac{{\text{π}}^{4}\mu_{i}L_{i}}{8h_{i}^{3}w_{i}\left(1-\frac{2h_{i}}{\text{π}w_{i}}\tanh\left(\frac{\text{π}w_{i}}{2h_{i}}\right)\right)}$$

$P_{\text{drag}}/Q$ = viscous drag force (flow resistance) [N]

$\mu_{i}$ = viscosity of fluid i [Pa s]

$L_{i}$ = length of channel i [m]

$h_{i}$ = height of channel i [m]

$w_{i}$ = width of channel i [m]

Because the aqueous phase does not come into direct contact with surfaces in this SlipChip design, the loss of material to adsorption or sticking is minimized, which is important for applications where there is low input of materials. One such application is handling nucleic acids originating from small numbers of, or even single, cells.

**Fig. 2:**
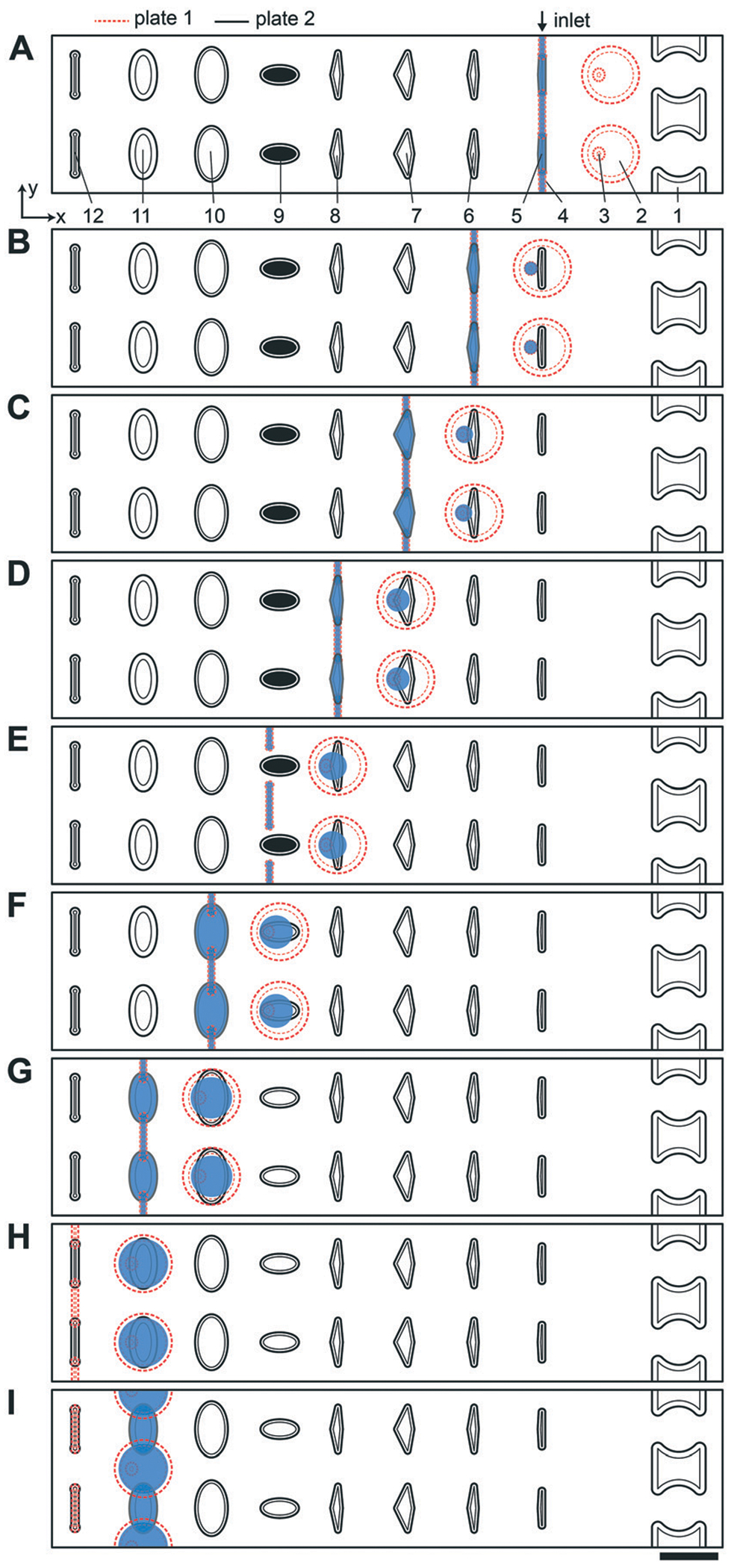
Top-down view of two-row section of the multistep SlipChip device. (A) The two plates of the multistep device are assembled and aligned. Features and their corresponding depths: [1] evacuation channels (100 μm); [2] mixing wells (100 μm); [3] surface-energy traps at the bottom of mixing wells (70 μm), mixing wells have a capacity of 71 nL together with the surface-energy traps; [4] connecting channels (40 μm); [5] carrier wells, 3 nL (50 μm); [6] lysis solution wells, 5 nL (50 μm); [7] RNA 3’-end repair solution wells, 7.4 nL (50 μm); [8] denaturing agent loading wells, 5.4 nL (50 μm); [9] barcode wells (50 μm); [10] ligation mix wells, 20 nL (50 μm); [11] crowding agent wells, 28.6 nL (100 μm); [12] channels for optional clearing of the connecting channels shown in feature [4]. First set of wells (type [5]) is shown being loaded through the inlet. Inlet and outlet holes for loading/unloading are drilled in plate 1 (Fig. S1†). (B) A sample drop-in into mixing wells and subsequent loading of the lysis solution. (C) Drop-in of lysis solution into the mixing wells and heat-treating the device to lyse cells and fragment RNA. Loading of the RNA 3’-end repair solution occurs in the same conformation. (D) Drop-in of 3’-end repair solution and loading of the denaturing agent. (E) Drop-in of denaturing agent. (F) Addition of the pre-spotted barcodes and loading of the ligase solution. (G) Drop-in of the ligase solution and loading of the crowding agent solution. (H) Drop-in of the crowding agent. Optionally, the loading channels can be drained and reloaded with carrier fluid in this conformation. (I) Plate 1 is shifted up to form a channel out of overlapping mixing wells and crowding agent wells. Using this newly formed channel, the user can pool and extract the contents of the wells with a pipette. Scale bar: 1 mm.

We used the drop-in approach from Fig. 1 to design a device (Fig. S1†) to perform multistep transcript barcoding for multiplexed RNA sequencing library preparation, described in more detail below. Briefly, we wanted to test whether we could use the multistep device to facilitate sensitive recovery of barcoded RNA transcripts. As the starting point for the workflow, we selected and modified the published RNAtag-Seq method^35^ for total RNA sequencing (Fig. 2). To block/dilute the undesired components of preceding reactions (*e.g.*, to avoid inhibition in an additive protocol), we sequentially added reagents in a range of volumes that changed the composition of the reaction buffer between steps. Because this biochemical workflow required carrier wells of different volumes, we chose to use a sequential drop-in approach rather than the back-and-forth approach described in Fig. 1.

**Fig. 3:**
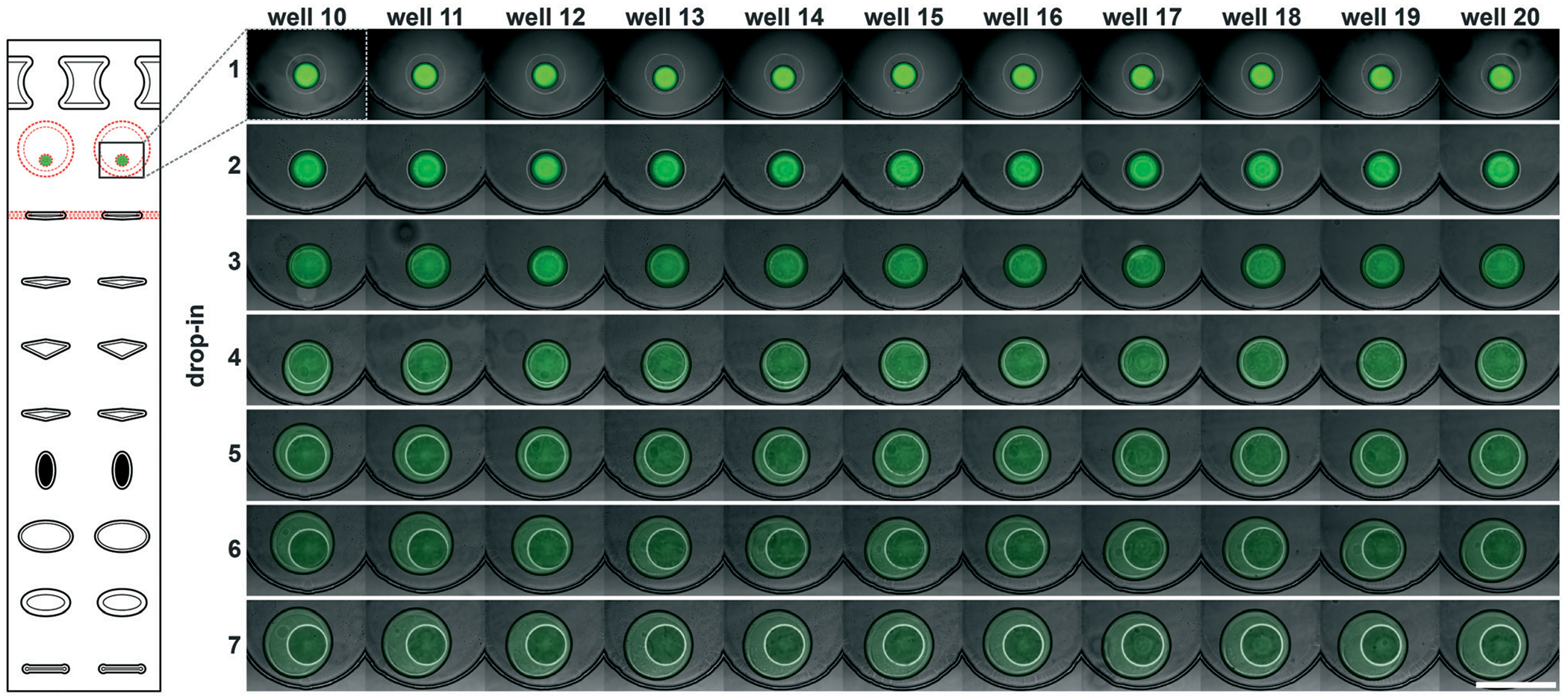
A subset of the multistep SlipChip device (wells 10–20) showing overlaid fluorescence and bright-field images after each of seven drop-in and merging events of 3 nL 50 μM Alexa Fluor 488 droplets. Scale bar: 500 μm.

**Fig. 4:**
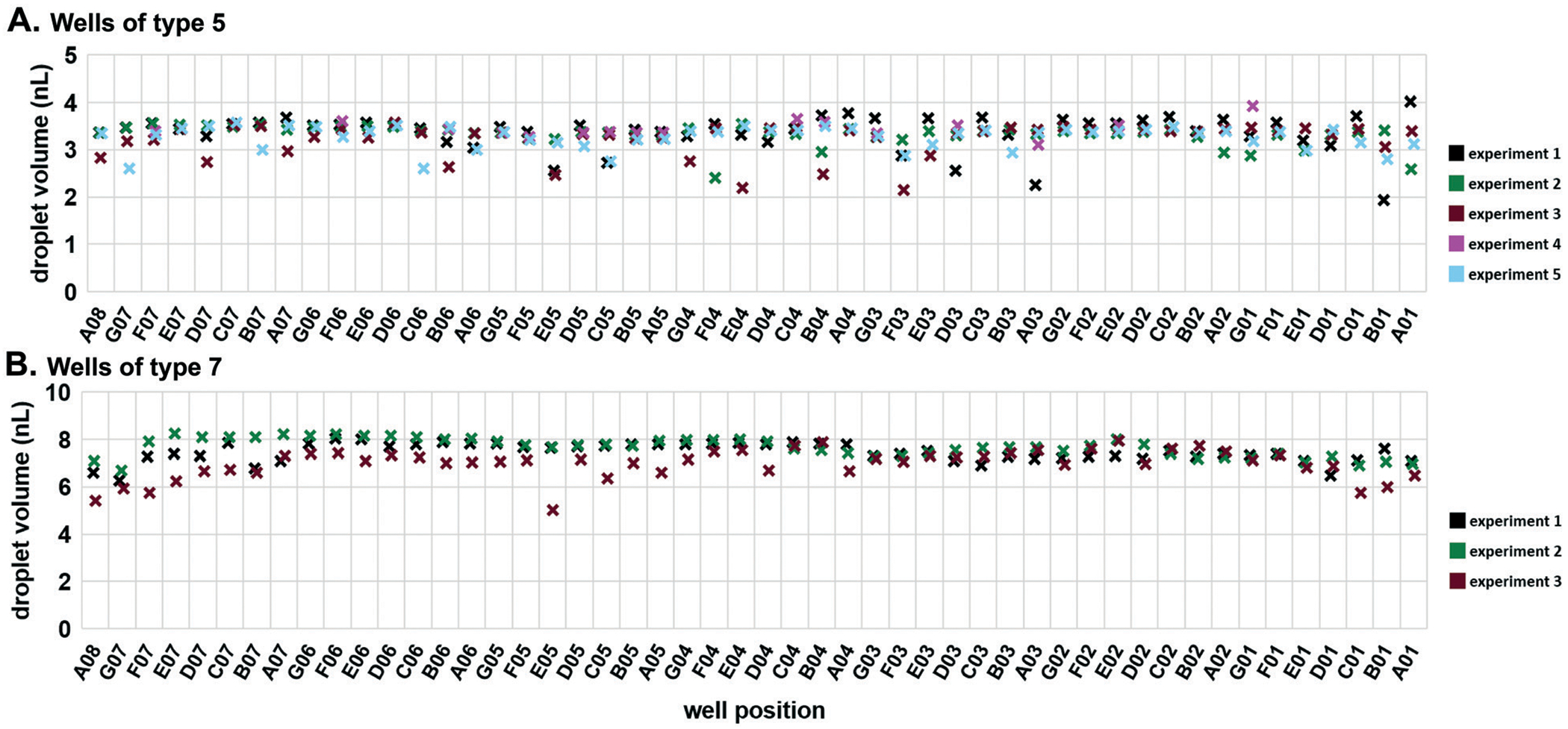
Reproducibility and spatial distribution of volume metering by carrier wells in the multistep SlipChip. (A) Wells of type 5, measured volumes of 3.30 ± 0.31 nL (mean ± S.D.); (B) wells of type 7 measured volumes of 7.36 ± 0.57 nL (mean ± S.D.).

**Fig. 5:**
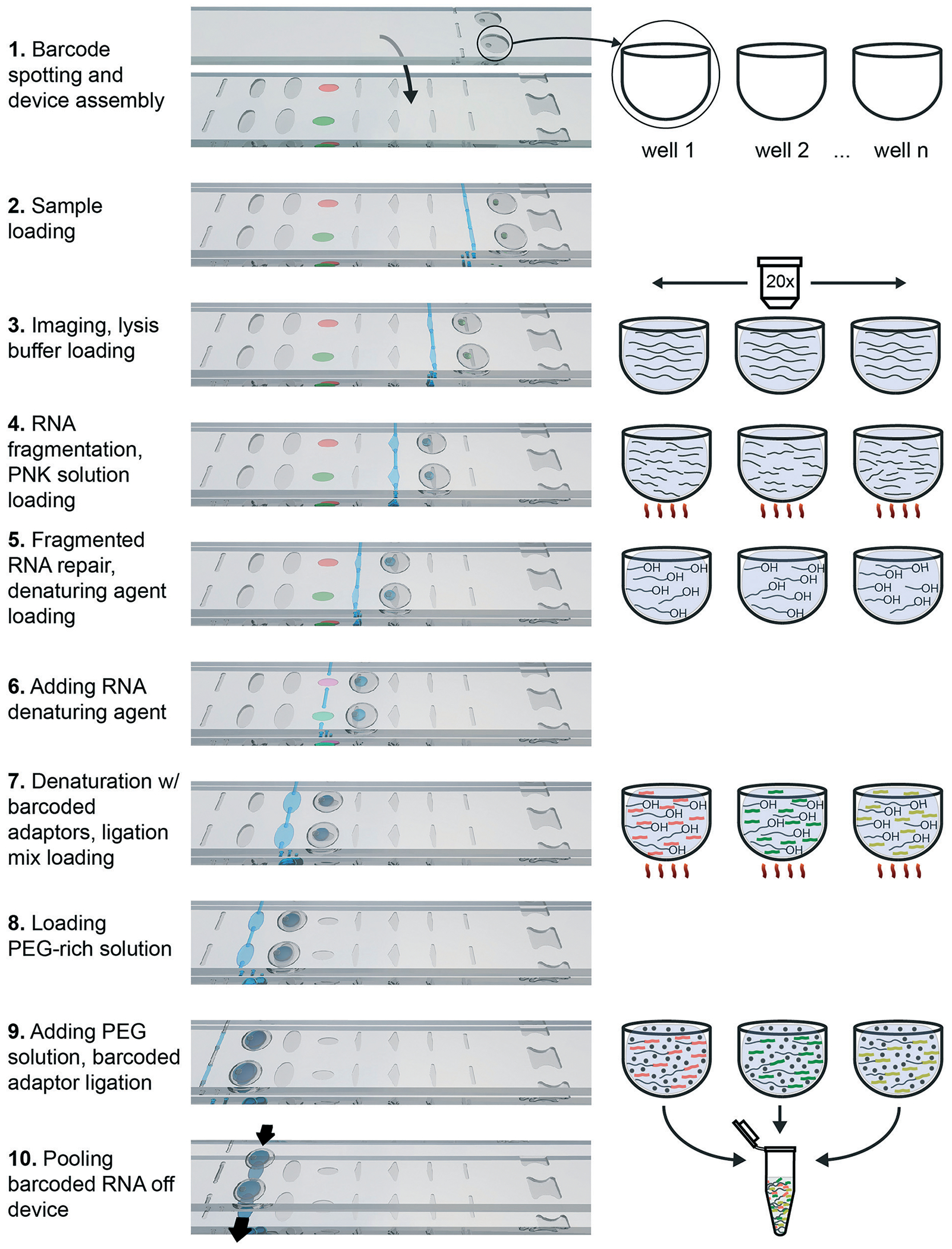
Three-dimensional rendering of a two-well row section of the multistep SlipChip and a schematic illustrating the fragment barcoding process. Black lines represent RNA, and orange/green/yellow fragments represent barcodes. PEG crowding agent is represented by black dots.

**Fig. 6:**
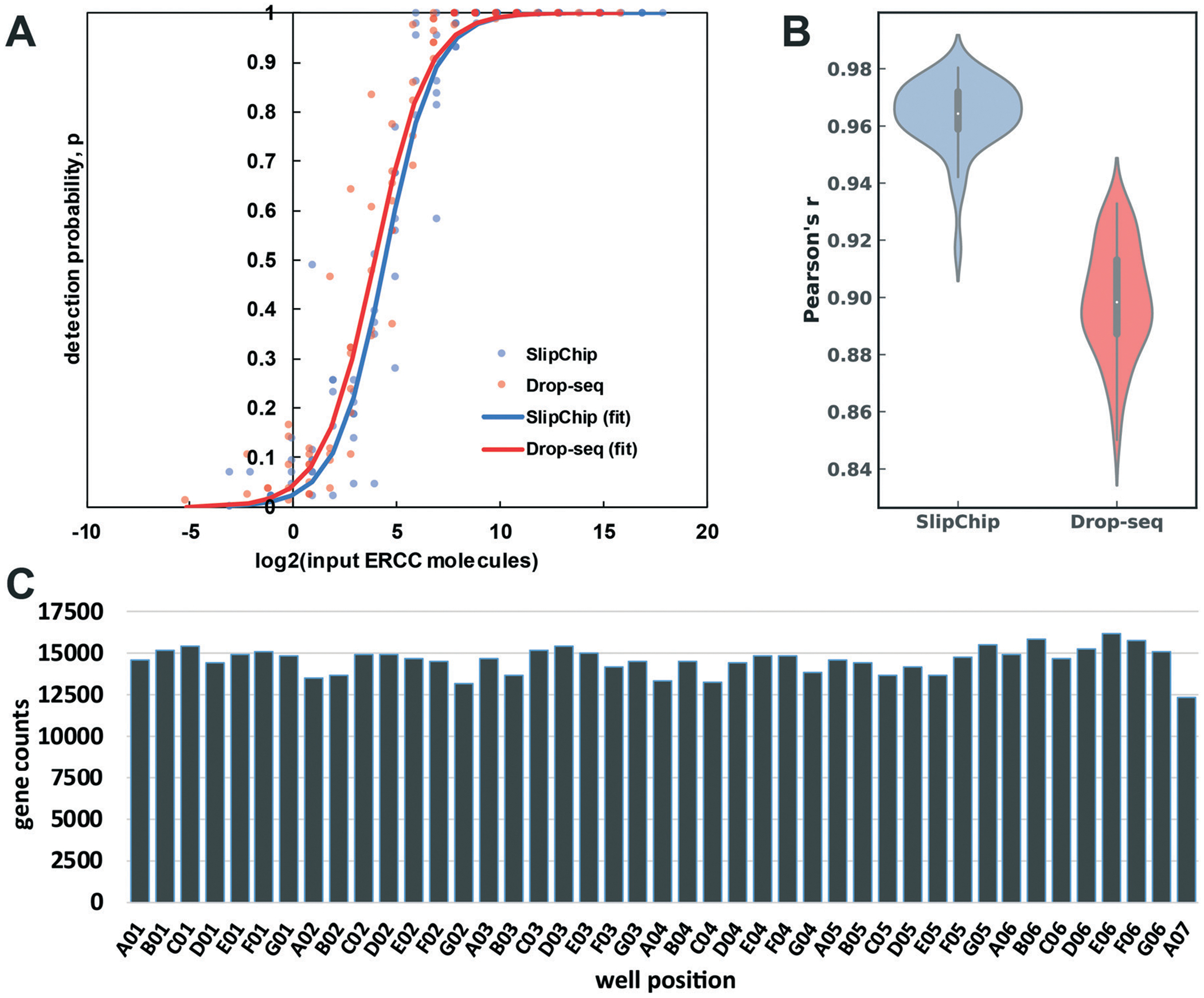
An overview of device-performance metrics. (A) To evaluate sensitivity, the ERCC transcript detection data was used to estimate binomial parameter *p* (detection probability) for various levels of input ERCC molecules. Logistic regression model was used to obtain a sigmoidal fit (eqn (3)). Based on these regressions, the multistep device required 21 molecules for 50% probability of detection, whereas the Drop-seq method required 15.^1^ (B) Distribution of Pearson correlation coefficient values (accuracy of quantification) between the detected expression units (TPM) of ERCC molecules and their input levels across the barcodes in our device and Drop-seq data set. The center of the box plot represents the median, thick gray line represents the interquartile range, and the thin gray line represents 1.5× interquartile range (the rest of the distribution, without outliers). (C) Gene count per barcode from repaired human RNA (loaded at 67.2 pg per well). Sequencing depth: 3.7 × 10^7^ paired-end reads (0.85 million reads per barcode). Barcodes are shown according to spatial placement within device.

In the multistep SlipChip device, the user first loads the sample (Fig. 2A-[4 and 5]). When the device is slipped (Fig. 2B), the sample is compartmentalized and transferred from the carrier wells (Fig. 2A-[5]) into the mixing wells (Fig. 2A-[2]). Note that the fluid remaining in the connecting channels (Fig. 2A-[4]) does not get transferred into mixing wells, which means that these connecting channels can be of arbitrary length (the spacing of carrier wells will have to match this length). For the device shown, we chose a length of 0.75 mm, which was convenient for manual slipping, while keeping the total footprint of the device low (Fig. S1†). The mixing wells (Fig. 2A-[2]) contain surface-energy traps (Fig. 2A-[3]) that help position the droplets for downstream reagent additions (these traps are explained in detail in the next section). The device is designed so that when it is slipped into the drop-in position for one carrier well, the device is also configured into the loading position for the next carrier well. For example, Fig. 2B represents the conformation for both dropping-in the sample from the first set of carrier wells (Fig. 2A-[5]), and for loading of the first reagent into the second set of carrier wells (Fig. 2A-[6]). The next three mixes of reagents are loaded, compartmentalized, and transferred into the mixing well in the same fashion (Fig. 2C–F) with the next three arrays of carrier wells (Fig. 2A-[6–8]). Note that wells of type 9 (Fig. 2A-[9]) each contain a different dry reagent spotted on the surface and therefore do not need to be loaded through the connecting channels (Fig. 2A-[4]). These wells are given an appropriate shape to avoid contact with these channels during slipping (as shown in the configuration Fig. 2E), and are made large enough for the user to be able to spot either manually or robotically. Once the pre-dried reagents are dissolved in the contents of the mixing wells, the next two reagent additions (Fig. 2F–H) are performed using the same drop-in principle as in Fig. 2A–E. Although there is not a strict rule on the shape of the carrier wells in the *XY*-plane, we observed that making them wider in the middle streamlines the drop-in process by “scooping” the fluid being carried inside the larger mixing wells towards the droplet anchored by the surface-energy trap (Fig. 2A-[3]), especially in the early steps of the protocol, when the volumes in the mixing wells are still small. Thus, we made the wells of types 10 and 11 (Fig. 2A-[10 and 11]) oval, whereas we made carrier wells of types 5–8 quadrilateral (Fig. 2A-[5–8]). Wells of type 11 (Fig. 2A-[11]) serve a dual purpose: they are carrier wells and they form a channel that connects the samples for pooling and extraction of the final products off-device (Fig. 2I). To speed up this pooling step and to maximize sample recovery, the wells of type 11 (Fig. 2A-[11]) were etched to the same depth as the mixing wells to reduce the flow resistance.

High viscosity continuous fluids have been reported to minimize the thinning and rupture of the wetting layer (which can lead to aqueous phase sticking) in SlipChip devices^27^ and have been shown to speed up mixing after coalescence of dispersed-phase droplets.^37,38^ We also observed that lower viscosity oils may wash away the pre-spotted reagents during device assembly as the extra oil is pushed out when the device plates are forced together (due to higher Reynolds number). Therefore, we chose to use 50 mPa s silicone oil with 0.01 mg mL^−1^ Span-80 for continuous phase. This fluid has been previously tested with dichlorodimethyl–silanized glass devices.^27^ A more viscous carrier fluid was undesirable because the flow resistance becomes too high during oil draining, and high viscosities have been demonstrated to slow droplet coalescence.^37,38^

### Device designs that enable drop-in

We next determined which device dimensions would enable drop-in of droplets in parallel. Some applications, especially those involving biology or time-sensitive reactions, require parallelized reagent addition in a single multiplexed experiment. In the case of RNAtag-Seq with live cells,^35^ one such example is the addition of the lysis buffer to all device wells, immediately before heating up the device to 72 °C for actual lysis (Fig. 2C). If this addition is performed unevenly across the device, the non-uniform exposure of the cells to non-ionic detergent and EDTA (without heat-killing them) could affect transcription. Another example is adding ligase mixture right after the RNA denaturation step (Fig. 2F). Denaturation is performed at 65 °C and the device needs to be immediately cooled on ice. The ligase mixture should be added while the device is still cold, so the 3′ and 5′ ends of the nucleic acids do not recover their secondary structures before they are stabilized by the ligase.

We selected a combination of 50 μm and 100 μm well depths to test drop-in. These depths produced convenient dimensions and the nanoliter-scale volumes desired for our miniaturized reactions. Even though the densities of our continuous and dispersed phases are slightly different (0.96 g cm^−3^ and 1.0 g cm^−3^, respectively), capillary forces dominate over gravity at these dimensions,^39^ and drop-in works regardless of the relative orientation of plates 1 and 2. Our goal was parallel merging across the device, and although we saw successful droplet transfer with these dimensions, merging did not take place in all wells in parallel. Because the droplets are much lower in volume than the volume capacity of the mixing wells, the droplets to be merged may not always be in contact inside the mixing wells. Merging across all wells can be achieved by additional slipping of plates, however we wanted a fast drop-in with a narrow distribution of merging times.

To accelerate drop-in and achieve uniform merging, we integrated two features that control droplet position during slipping and inside the mixing wells. To localize the droplets inside the mixing wells, we equipped these wells with surface-energy traps (Fig. 2A-[3]).^32–34^ These traps are auxiliary wells that provide increased dimensions that allow lower surface-energy conformations of the droplets, thus anchoring them. We tested several depths and locations of the traps. We made the traps 70 μm at the bottom of the 100 μm mixing wells because an initial droplet of 3 nL will have a diameter of ~180 μm^3^ if allowed to assume a spherical shape. We also observed that placing the trap closer to the mixing well drop-in side enhanced merging (Fig. 2A-[3]). Importantly, with the surface-energy traps, the droplets are in a regularly spaced array (Fig. 3), which allows us to automate imaging.

Occasionally, a carrier well may not be loaded to capacity; in such cases, it is preferable for the well to contain a single volume (instead of multiple unmerged droplets) to facilitate further merging. To minimize the chance of droplet breakup during slipping, we made the carrier wells wider in the middle (Fig. 2A-[5–8]). This way, smaller droplets are scooped toward the middle of the carrier wells during slipping and are aligned with the surface-energy traps of the mixing wells. Using microscopy, we confirmed that the multistep drop-in worked—each device slip transferred a new droplet from carrier well into each mixing well (Fig. 3).

### Additional device features

To enhance volumetric metering, we introduced a slight difference in depth between the connecting channels (Fig. 2A-[4]) and carrier wells (Fig. 2A-[5]). In loading conformation, the fluidic path is made of carrier wells in one plate and connecting channels in the opposite plate, in an alternating sequence. When an aqueous sample is loaded, it fills both the carrier wells and the connecting channels. Only the volumes filling the carrier wells will be transferred into the mixing wells. To ensure carrier wells are fully filled, we made the connecting channels shallower than the carrier wells (40 μm *vs.* 50 μm). Driven by capillary pressure (the same principle that enables the drop-in approach), the non-wetting aqueous phase will preferentially occupy the deeper carrier wells (Fig. 1). This driving force is positively correlated with the difference in depth between the two features and can be estimated by using eqn (1).^27^ However, the flow resistance is inversely proportional to the cube of channel height (eqn (2)),^27,36^ and we observed that making the connecting channels less than 40 μm resulted in prohibitively high resistance to flow with the oil viscosity that we used (50 mPa s).

To test the ability of these geometries to yield reproducible drop-in and merging, we made a device according to the specifications in Fig. 2, and used it to merge arrays of 50 3 nL droplets seven times using a back-and-forth slipping motion (moving between the conformations seen in Fig. 2A and B). We observed successful droplet merging, which resulted in regular arrays of droplets after each addition (Fig. 3).

We next added the ability to unload and reload samples during a single experiment. In addition to the obvious need for extracting the mixing-well contents from the device at the end of the procedure, we wanted to be able to restart an experiment without having to re-assemble and re-spot the device. For example, applications where samples are loaded before imaging with only a crude estimate of concentration (*e.g.* cell cultures), the user may unknowingly over- or under-load the device. In this case, it would be advantageous to be able to image and re-use the device without proceeding with the entire experiment. Evacuation channels (Fig. 2A-[1]) provide the user the option to flush the contents of all mixing wells at any step before addition of the barcoded adaptors into the mixing wells (Fig. 2F). By slipping device plate 1 in the opposite direction from the carrier wells of plate 2, the mixing wells can be aligned with the evacuation channels to form a continuous channel (Fig. S2C†). In this conformation, the mixing wells can be emptied out with a pipette and re-filled with oil.

Next, we tested the reproducibility of volumetric metering in the device to confirm there was no spatial bias during loading. Deviations in the volumes delivered to mixing wells are due to errors in the loading of the carrier wells. To assess this variability, we loaded the device with 50 μM Alexa Fluor 488 (#A33077; Thermo Fisher) solution and slipped the carrier wells away from the connecting channels without dropping the contents into the mixing wells. We imaged these droplets using confocal microscopy and used the *Z*-stacks to calculate the droplet volumes (Fig. S3†). Using two different well sizes, we confirmed reproducible loading, both among wells within a single device, and among replicate trials (Fig. 4). The coefficients of variation were of similar values for both well types: 0.093 for type 5 (Fig. 4A) and 0.077 for type 7 (Fig. 4B).

### Demonstration of the multistep device using RNA barcoding for RNA-Seq

To demonstrate the functionality of this device in performing complex biochemistry protocols, we chose to use it for RNA barcoding for multiplexed RNA sequencing (RNA-Seq). RNA-Seq is becoming an increasingly popular technology for transcriptomic studies, and any improvement in the sensitivity, accuracy and applicability for new types of samples would be of great benefit to the field. The published RNAtag-Seq method allows barcoding through direct ligation of RNA adaptors to fragmented and repaired total RNA. It is a strand-specific and full-length transcript-detection method that may be applied to any type of RNA, including RNA of prokaryotic and eukaryotic origins. We modified the barcoding protocol from the published RNAtag-Seq method to perform it in an additive fashion (as described in ESI†). We specifically selected an extraction method that works for single eukaryotic cells and for Gram-negative bacteria^40^ and incorporated this method into an additive biochemical protocol that provides compatible conditions for barcoding by ligation downstream.

The protocol consists of four sequential biochemical reactions: (i) template RNA fragmentation on-device under RNA extraction conditions; (ii) template RNA fragment ends repair; (iii) barcoded RNA adaptors & template RNA fragments denaturation; and (iv) ligating barcoded RNA adaptors to template RNA in every device well. These reactions were performed in a 7-step device workflow (Fig. 5), starting from loading RNA (Fig. 5-2) and ending by pooling the barcoded transcripts from device (Fig. 5-10). As a result, we were able generate and pool multiple barcoded intermediates for the subsequent RNA-Seq libraries preparations in a single device workflow.

Briefly, the barcoded RNA adaptors are spotted on plate 2 of the device and dried in the presence of trehalose before device assembly with silicone oil (Fig. 2A-[9] and 5-1). Next, the sample containing RNA of interest is loaded into the device (Fig. 2A and B and 5-2 and 3) through the drilled holes with a pipette and slipping the plates along *x*-axis. RNA can be loaded in Tris-EDTA buffer, water, PBS, or cell culture media. We then add a lysis buffer that contains EDTA and non-ionic detergents to mimic conditions of live cell lysis^40^ and fragment RNA by heat (2 min at 72 °C, followed by an optional 3 min at 91 °C) (Fig. 2B and C and 5-3 and 4). Next, T4 polynucleotide kinase (T4 PNK) is combined with the reaction mixture (Fig. 2C and D and 5-4 and 5), it is added in the buffer that provides the optimal salts concentration in resulting reaction volume. T4 PNK removes occasional phosphates from the 3′ end of the RNA fragments. After the repair reaction, DMSO is added as an RNA denaturing agent (Fig. 2D and E and 5-5 and 6), and the pre-spotted barcodes are combined with the repaired RNA fragments in the mixing well (Fig. 2F and 5-7). Heating the device to 65 °C denatures RNA and inactivates T4 PNK. The last two steps are adding the T4 RNA Ligase (Fig. 2F and G and 5-7 and 8) and a crowding agent solution (Fig. 2G and H and 5-8 and 9). After allowing the ligation reaction to take place overnight, the contents of all mixing wells may be pooled off-device by slipping the plates along the *y*-axis and connecting the mixing wells (Fig. 2A-[2]) and crowding agent wells (Fig. 2A-[11]) to form a continuous channel for sample pooling (Fig. 2I and 5-10). The contents of the device can now be extracted through drilled holes with a pipette. The rest of the cDNA library preparation takes place in a single tube, in a protocol similar to the published bulk RNAtaq-Seq method (see ESI†).^35^

To test the accuracy and sensitivity of this workflow in our device, we used it to barcode a dilution of External RNA Controls Consortium (ERCC) transcript spike-in kit, a standard tool used in benchmarking of RNA-Seq methods (Thermo Fisher, Waltham, MA). After preparing and sequencing the cDNA library to approximately 0.6 million paired-end reads per barcode, we used published metrics^41^ to evaluate the sensitivity (detection limit) (Fig. 6A) and accuracy (Fig. 6B) of our transcript quantification. We compare it to the published Drop-Seq method, which was sequenced to 2 million reads per barcode.^1^ Our observed sensitivity and accuracy are on par with or out-perform other published methods (Fig. 6A and B).^41,42^ The detection limit was 21 copies (the input level with detection probability >0.5), which is a competitive result at this sequencing depth, and is comparable to the performance of other methods (Fig. 6A).^41,42^ We believe this number can be brought even lower by increasing sequencing depth because in a comparison of RNA-Seq methods, sensitivity was more responsive than accuracy to sequencing depth.^41^

To assess the quantification accuracy, median Pearson correlation coefficient between log2 (input molecules) and log2 (quantified expression values in transcripts per million (TPM)) was computed to be 0.96, which out-performs most competing barcoding methods.^41^ We compared our accuracy to a 84 barcode data set published in 2015 by Macosko *et al.*,^1^ with a median Pearson correlation coefficient value of 0.90 (Fig. 6B).

We wanted to check for uniformity of gene detection across wells by loading the device with extracted RNA. We loaded repaired total human RNA at a concentration of 67.2 pg per well (equivalent to the RNA content of a large mammalian cell^43^) and barcoded it on-device. Next, we pooled the device contents and generated the cDNA library in a single tube. We sequenced this library to 0.85 million reads per barcode (Fig. 6C), which produced 13 182 ± 514 (mean ± S.D.) feature counts per well. These results are encouraging at this sequencing depth^44–46^ and importantly, we observed no spatial bias on-device in the distribution of reads among barcodes (Fig. S4†).

## Experimental section

### Device fabrication

We used 700 μm thick soda-lime glass plates to fabricate the devices. The plates were coated with 125 Å Cr/1000 Å Au/10000 Å AZ1500 photoresist (TELIC, Valencia, CA). The features were fabricated in the plates with standard multi-step photolithography protocols. Photolithography masks (see ESI†) were designed in AutoCAD (Autodesk, San Rafael, CA) and printed by CAD/Art Services, Inc. (Bandon, OR). The volumes of features were also calculated, based on isotropic etching modeling in AutoCAD. Holes for loading/unloading were drilled in the plate with the mixing wells with a diamond drill bit (0.035″ diameter; Harvey Tool, Rowley, MA). The glass plates were then plasma treated and gas-phase silanized with dichlorodimethylsilane (Sigma, Cat.# 440272), as described in previous works.^26^

### Reagent pre-spotting

For pre-spotting of oligo barcodes, we used trehalose solution (100 nL droplets of 20 mM trehalose), for its documented nucleic acid stabilization properties^47^ as well as adhesive properties. Once dried for at least 15 min, trehalose-containing droplets result in sticky semi-crystalline spots that securely stay in wells during device assembly. Spotting was performed with automated BIODOT AD2000 aspirate/dispense platform (BIODOT, Irvine, CA), but may also be done by manual pipetting.

### Device assembly

The plates were assembled by pipetting 0.5 mL of 50 cSt silicone oil (Clearco Products, Willow Grove, PA) on one of the plates and sandwiching the oil with the other plate. Aligning and slipping was performed manually under 2× magnification.

### Device operation

Prior to loading, the oil was drained from an aligned fluidic channel by applying negative relative pressure at the device outlet. This was done by either a micropipette or with a syringe with an attached micropipette plastic tip. Sample loading was performed with a micropipette at the device inlet, which can be sped-up by using a second pipette at the outlet to increase the pressure difference between inlet and outlet. This device may be slipped either before removing pipettes or after. Alternatively, the user can remove one pipette, slip one side, then removing the second pipette, and slip the other side of the device.

### Epi-fluorescence imaging of drop-in/merging

Imaging of 50 μM Alexa Fluor 488 dye solution (Thermo Fisher, Waltham, MA) was performed on Leica DMI6000B with Leica A8 automated stage (Leica Microsystems, Buffalo Grove, IL), interfaced with MetaMorph software (MetaMorph, Nashville, TN).

### Confocal microscopy for metering quantification

Imaging of 50 μM Alexa Fluor 488 dye solution (Thermo Fisher, Waltham, MA) was performed on Zeiss LSM 800 interfaced with ZEN 2 blue edition software (Zeiss, Oberkochen, Germany). Up to 20× magnification was used, but higher resolution possible with long working distance objectives. Calculation of volumes was performed using Imaris suite v9.1.0 (Bitplane, Concord, MA) (Fig. S3†). The smooth/surface detail was set at 4 μm, and the absolute intensity threshold was selected automatically by the software.

In our analysis of the reproducibility and spatial distribution of volume metering (Fig. 4), we investigated statistically the uniformity of volume metering in the edge wells. We used a two-tailed Welch’s t test (*α* = 0.05). For wells of type 5, the volumes of the edge wells (A08 and A01) were not significantly different from the volumes of the type 5 wells (*P*-values of 0.591 and 0.947, respectively). In wells of type 7, the volumes of the edge wells (A08 and A01) were also not statistically different from the rest of the type 7 well volumes (*P*-values of 0.179 and 0.114, respectively).

### Using device for barcoding RNA transcripts (ERCC transcripts and repaired human RNA)

The device that was used for this validation was a prototype with 43 barcoded wells. Mix 1 of ERCC transcripts (Thermo Fisher, Cat.# 4456740) was diluted 220 times in PBS and dropped-in into the mixing wells using 3 nL carrier wells (this corresponds to 850 067 transcripts per well). Lysis buffer was added using 5 nL carrier wells (no heating steps were performed at this point). RNA fragments repair solution was added with 8 nL carrier wells, followed by 30 minute incubation at 37 °C. RNA denaturing agent dimethyl sulfoxide (DMSO) solution was added next in 5.4 nL carrier wells, and the pre-spotted ssRNA adaptors with barcodes (with blocking groups on their 3′ ends) in the next set of carrier wells were dissolved in the reaction mix. At this point, the device was incubated at 65 °C for 2.5 min to melt any secondary RNA structures, then immediately placed on ice. Next, T4 RNA Ligase solution was added in the 20 nL wells, followed by an addition of PEG solution in the 28.6 nL wells. The device was incubated at room temperature for at least 4 hours. After ligation, the device was frozen and stored at −20 °C before pooling samples. To pool barcoded nucleic acids the device was thawed and slipped to the pooling position (Fig. 2I) and the contents were collected with a pipette. To maximize recovery, the channel was washed two times with 10 μL of wash buffer containing EDTA (which chelates Mg^2+^ ions and stops enzyme activity), non-ionic detergent and RNAse inhibitor. The eluent of these wash steps was combined with the pooled sample for subsequent off-device steps.

For the experiment with human total RNA (Fig. 6C), we loaded water in 3 nL carrier wells and lysis buffer in 5 nL carrier wells to mimic lysis conditions. Next, repaired total human K562 RNA was loaded to device at 67.2 pg per well in 8 nL wells in RNA fragments repair solution. The heating steps were omitted for this experiment, but the rest of the protocol was carried out as described above. The rest of the cDNA library preparation for 1 and 2 took place in a single tube, in a protocol similar to described in RNAtaq-Seq method,^35^ starting from step 4, with some modifications (see ESI†). Illumina paired-end sequencing was performed using 34 bp-long read 1 and 36 bp-long read 2.

### Sequencing data analysis

Sequence alignment was performed using STAR (v. 2.6.1b) with default settings (option – alignIntronMax was set to 1 for ERCC spike-in reads). Human RNA reads were aligned to RefSeq human genome assembly GRCh38. UMIs were deduplicated and counted using umi-tools package (v. 0.5.5). Features were counted using featureCounts package (v. 1.6.3). For logistic regression model for sensitivity evaluation we used MATLAB built-in function binofit to fit the transcript-detection data as binomial trials and applied the regression method from Svensson, *et al.* (2017) to estimate parameters *a* and *b* to obtain the detection limit (Fig. 6A):^41^

(3) $$P\left({\text{detected}}_{i}\right)=\frac{1}{1+{\text{e}}^{-\left(a\times \log_{2}\left(M_{i}\right)+b\right)}}$$

where $M_{i}$, is the number of ERCC molecules i spiked in and where the detection limit = $2^{-\frac{b}{a}}$.

For dose–response Pearson correlation coefficient for accuracy evaluation the de-duplicated reads (UMI counts) were converted into normalized units of expression of TPM and Pearson correlation coefficient between log2 (TPM) and log2 (input ERCC molecules) was calculated for each barcode (Fig. 6B).

## Conclusions

We conceived and validated a device that combines several physical phenomena to provide a simple tool for encapsulation, imaging, and additive protocol execution in nanoliter-scale wells. The multistep SlipChip device allows the user to make repeated and complete droplet transfers that enable multistep processes with the option to retrieve the final products. The key innovation that was foundational to this technology was using the dimensions of device features to shape the interface between two immiscible fluids to facilitate droplet transfer. By adding geometric enhancements we also improved reproducibility of the droplet merging. Importantly, the operation of the device does not require equipment other than micropipettes and a basic microscope, which makes it an attractive option for many applications.

To illustrate the capabilities of this device we carried out a complex, 4-part biochemical scheme: transcript barcoding for multiplexed RNA-Seq library preparation. Using standard benchmarking metrics^41^ to assess performance, we found that our method for transcript quantification had competitive sensitivity and better accuracy than many existing techniques. Given the multistep device’s high performance, we envision that it will be well-suited for single-cell analyses or studies with small numbers of cells. The stochasticity of encapsulation (Poisson loading) will be advantageous to applications with arbitrary cell sizes and shapes. Moreover, because RNAtaq-Seq is an all-inclusive barcoding method, performing this method on the multistep device can be used to study gene expression in prokaryotes.^35^ Other features of the multistep device that we believe will be advantageous to biological studies include its optical clarity for high-resolution imaging of loaded specimens and its thin construction for rapid and uniform temperature control, including freezing and thawing.

For applications where higher level of multiplexing is desired, the device can be expanded to have additional wells, or multiple devices can be used together. The devices tested in this work were multiplexed by up to 50 wells. To add more wells, the footprint can be compressed (*e.g.* features can be placed closer together), which may require higher-precision device slipping than manual operation can provide (*e.g.* by using a micromanipulator stage or by adding guiding features for manual slipping). Higher number of wells can also be achieved by increasing the total area of the device. In this case, the user may want to use carrier fluids of lower viscosities to reduce flow resistance in longer channels.

This technology is not limited to the surface chemistry and fluids described in this work. We demonstrated the addition of multiple liquids and a pre-spotted solid reagent, but this device can also be used to meter gas-phase reagents. It is worth noting that differences in droplet sizes and surfactant contents, as well as carrier fluid viscosities, can be modulated in this platform to further enhance droplet coalescence and mixing.^37,38,48^

In addition to single-cell RNA-Seq and other single-cell assays, we anticipate this technology to be useful in other applications that benefit from multistep processing on a nanoliter scale, such as single-molecule assays, cell–cell interaction studies, clonal micro-colony studies, combinatorial approaches to protein crystallization,^49^ titrations, chemical synthesis, synthesis of monodispersed particles^50–52^ and heterogeneous colloidal assemblies,^53,54^ kinetics studies, batch and semibatch nanoliter reactors, and diagnostic assays.

## Supplementary Material

OA supplement

## Data Availability

Any data not in the ESI† will be made available upon request to the corresponding author.

## Footnotes

† Electronic supplementary information (ESI) available. See DOI: 10.1039/c9lc00541b
