# Supplementary material for: Microfluidic SlipChip device for multistep multiplexed biochemistry on a nanoliter scale
Source: Lab Chip. Author manuscript; Available in PMC 2024 Nov 5. (PMC11537478; doi:10.1039/c9lc00541b)
Supplement: OA supplement [file NIHMS2028909-supplement-OA_supplement.pdf]

Microfluidic SlipChip device for multistep multiplexed biochemistry on a nanoliter scale

Dmitriy V. Zhukov,[a] Eugenia M. Khorosheva, [a] Tahmineh Khazaei, [b] Wenbin Du,[c†]

David A. Selck, [a] Alexander A. Shishkin,[b] and Rustem F. Ismagilov[a,b]*

[a]Division of Chemistry and Chemical Engineering, California Institute of Technology 1200 E. California Blvd., Pasadena, CA, 91125 United States
[b]Division of Biology and Biological Engineering, California Institute of Technology 1200 E. California Blvd., Pasadena, CA, 91125 United States
[c]Department of Chemistry and Institute for Biophysical Dynamics, The University of Chicago, 929 East 57th Street, Chicago, Illinois 60637, United States
[†]Current address: State Key Laboratory of Microbial Resources, Institute of Microbiology, Chinese Academy of Sciences, Beijing, 100101 China

* Correspondence to: rustem.admin@caltech.edu

Figures S1-S4

Supplementary Materials and Methods

Differences and modifications to RNAtag-Seq protocol

Reagents for on-device steps

Supplementary References

Contributions of Non-Corresponding Authors

DWG photolithography masks for the multistep SlipChip (provided as .dwg file)

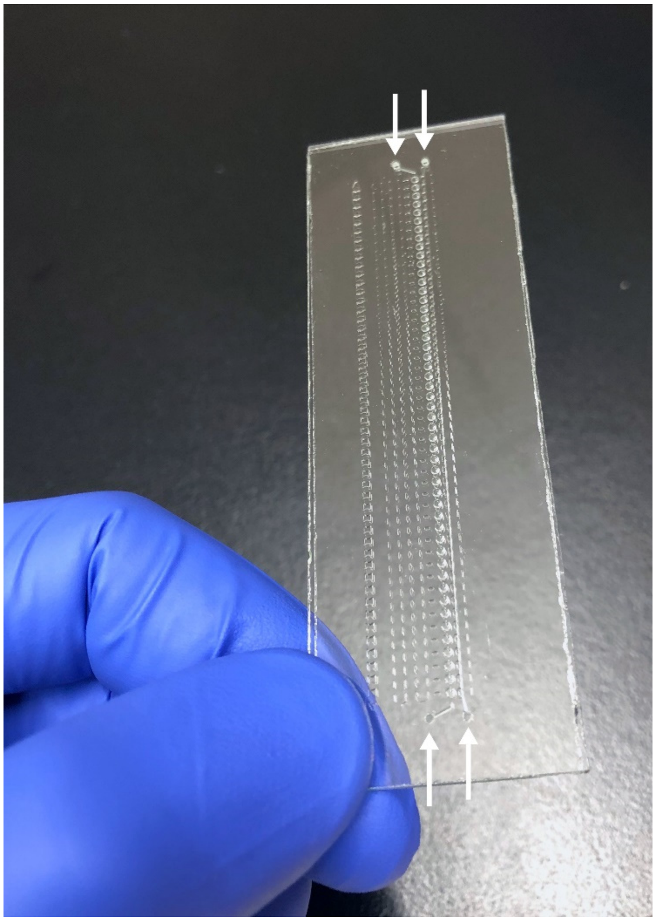

Figure S1: Photo of the multistep SlipChip. The arrows indicate drilled holes for device inlets and outlets. The footprint of the device is 27.4 x 82.9 mm. The .dwg file with photolithography masks for fabrication of the device is provided in the Electronic Supplementary Information.

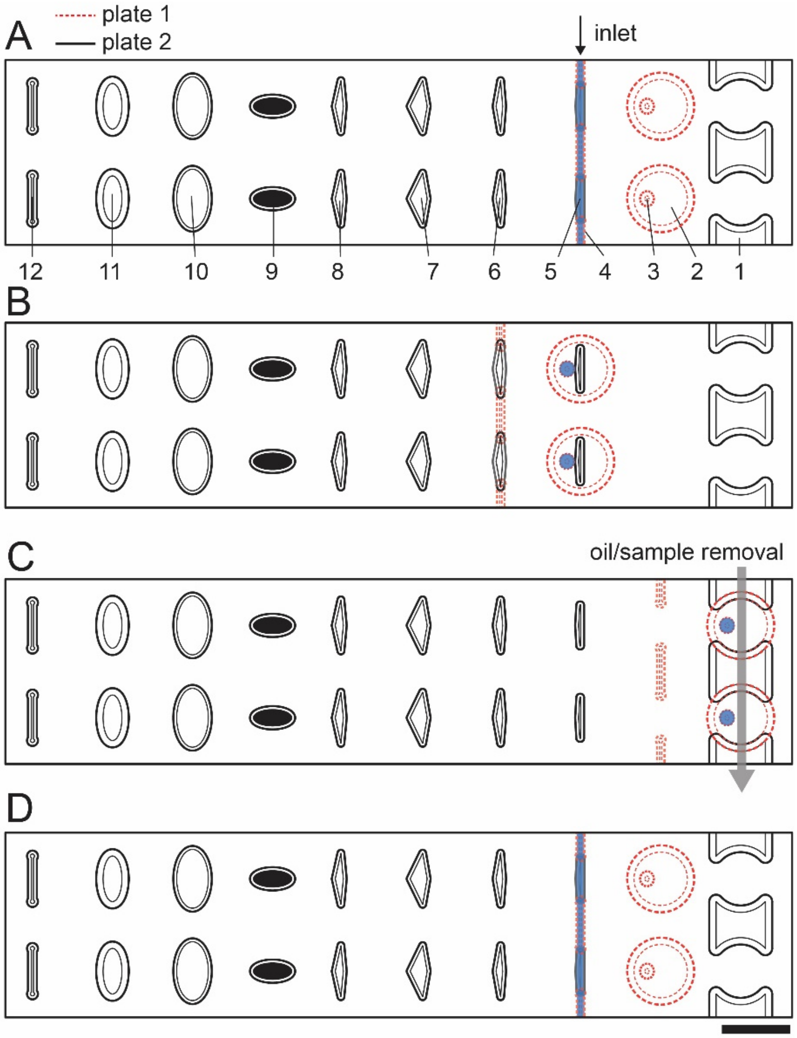

Figure S2: Top-down view shows the two-row section of the multistep device performing evacuation of the loaded mixing wells. (A) The multistep device is made of two plates, shown assembled and aligned. Features [1-12] and their corresponding depths: [1] evacuation channels (100 µm); [2] mixing wells (100 µm); [3] surface energy traps at the bottom of mixing wells (70 µm), mixing wells have a capacity of 71 nL together with the surface energy traps; [4] connecting channels (40 um); [5] sample wells, 3.3 nL (50 um); [6] lysis solution wells, 5 nL (50 um); [7] RNA 3'-end repair solution wells, 7.4 nL (50 um); [8] denaturing agent loading wells, 5.4 nL (50 um); [9] barcode wells (50 µm); [10] ligation mix wells, 20 nL (50 µm); [11] crowding agent wells, 28.6 nL (100 µm); [12] channels for optional clearing of the connecting channels [4]. First set of wells [5] is being loaded through the via holes. Via holes for loading/unloading are drilled in plate 1 (not shown). (B) Sample drop-in into mixing wells. In this configuration, the user can image the loaded samples to make sure under- or over-loading did not occur. (C) To restart the experiment, the device needs to be slipped into this conformation and the previously loaded sample pipetted out together with oil through the channel formed by connected mixing wells [2] and evacuation channels [1]. Channels [1] were shaped to minimize footprint while still fully overlapping with wells [2] during evacuation. Making the depth of the evacuation channels similar to the depth of the mixing wells prevents the sample droplets from being left behind in the deeper parts of the well-channel fluidic pathway. Fresh oil needs to be reloaded before the device is returned to conformation A. (D) the device is returned to the same starting conformation as A, and the new sample can be loaded. Scale bar: 1 mm.

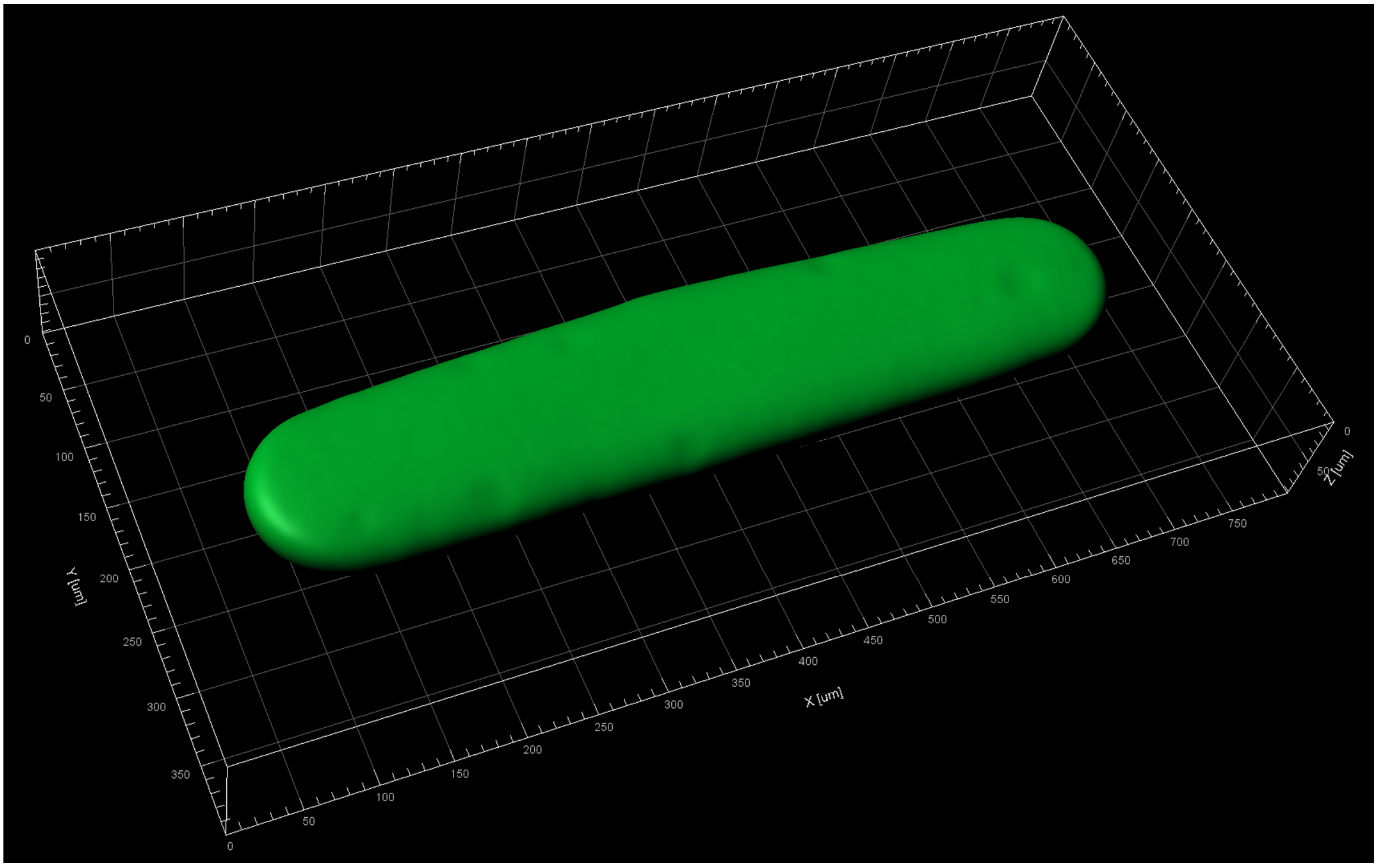

Figure S3: A three-dimensional reconstruction of a droplet inside a 3-nL well from confocal Z-stack for volume calculation in Imaris.

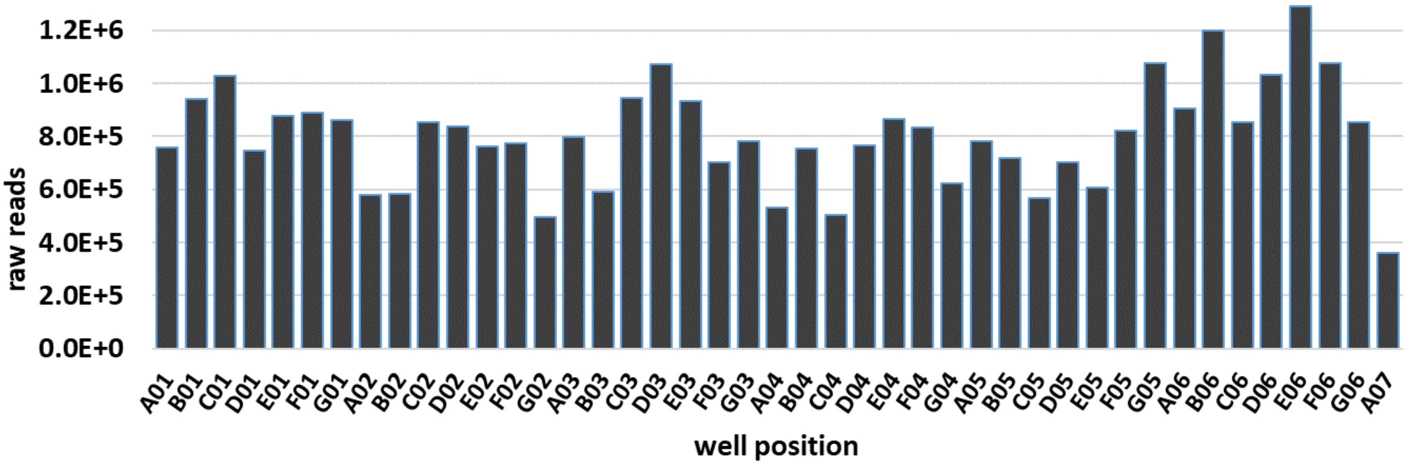

Figure S4: Spatial distribution of barcoded reads within the device for repaired total human RNA experiment. Reads per well that received a barcode: 802,812 ± 194,210 (mean ± S.D.).

Supplementary Materials and Methods

Differences and modifications to RNAtag-Seq protocol

1. UMIs (NNNNNNNN) were added to the RNA fragments during the second ligation.
2. rRNA depletion step was skipped.
3. Superscript IV (Invitrogen, Cat.# 18090050) enzyme was used for reverse transcription (RT).
4. RT primer (p14) was one base shorter than the published AR23 oligo:
   5´–/5SpC3/CTACACGACGCTCTTCC –3´ (IDT) was used for RT.
5. Second adaptor for ligation (p38) had UMI, C3 spacer and 5' phosphate as follows:
   5'–/5Phos/ANNNNNNNNAGATCGGAAGAGCACACGTCT/3SpC3/–3' (IDT).
6. We used Dynabeads MyOne Silane magnetic beads (Life Technologies, Cat.# 37002D) instead of using Zymo columns and RNAClean XP beads. Final concentration of EtOH in the mixture of RLT buffer (Qiagen, Cat.# 79216), RLT-washed Dynabeads MyOne Silane magnetic beads, and samples was 60% to clean up after the first linker ligation and RT step, and 70% of the final total volume after the second ligation.
7. Q5 Hot start High Fidelity DNA polymerase (NEB, 2XMasterMix, Cat.# M0494S) was used for the library PCR amplification with Illumina primers. Primer annealing was performed at 68°C for the first 3 cycles and at 70°C for the rest of cycles (18). The number of PCR cycles with Illumina primers was further adjusted: after initial amplification for 21 cycles we visualized libraries on agarose gel by gel electrophoresis using 1% E-gel EX (ThermoFisher, Cat.# G401001). To remove artifacts and Illumina adaptor dimers (that were major products of amplification at that stage) we cut gels to collect fragments between 200 bases and 800 bases. Next, we extracted libraries from gel using Zymo Gel Clean DNA recovery kit (Zymo Research, Cat.# D4002), and subjected them to additional amplification to compensate for dilution and added 3 more cycles. To remove artifacts and Illumina primers after the amplification we cleaned the libraries using 0.9% AmPure XP SPRI beads (Beckman Coulter, Cat.# A63880) twice.

Reagents for on-device steps

- **RNA samples** (added to mixing wells with 3 nL carrier wells):
  - Diluted in UltraPure DNase/RNase-free distilled water (Invitrogen, Cat.# 10977-015)
- **Lysis buffer** is based on the buffer used in Kang, et al. (2015)[1] (added to mixing wells with 5 nL carrier wells):
  - 100 mM Tris-Cl pH 8.0 (ThermoFisher Scientific, Cat.# BP1758-100)
  - 200 mM KCl (Invitrogen, Cat.# AM9640G)
  - 0.2 mM EDTA (OmniPur, Calbiochem, Cat.# 6381-92-6)
  - 0.1% Triton X-100 (Sigma, Cat.# T8787-50ML)
  - 2 mM DTT (Invitrogen, a tube from SuperScript IV kit, Cat.# 900147)
  - UltraPure DNase/RNase-free distilled water (Invitrogen, Cat.# 10977-015)
  - 0.5 U/µL SUPERase-In (Ambion, Cat.#AM2694; 2500U)
  - 1 µg/µL BSA (Roche, Cat.# 10711454001)
- **RNA fragments repair solution** (added to mixing wells with 8 nL carrier wells)**:**
  - 2 U/µL T4 PNK (NEB, Cat.# M0201S)
  - 2x T4 PNK buffer (NEB, Cat.# B0201S)
  - 0.25 mM $MgCl_2$ (NEB, Cat.# B9021S)
  - 0.5 U/µL SUPERase-In (Ambion, Cat.# AM2694)
  - 1 µg/µL BSA (Roche, Cat.# 10711454001)
  - UltraPure DNase/RNase-free distilled water (Invitrogen, Cat.# 10977-015)
- **RNA denaturing agent solution** (added to mixing wells with 5.4 nL carrier wells)**:**
  - 95% v/v DMSO (Sigma, Cat.# D2650)
  - 5% of 20 µg/µL BSA stock (Roche, Cat.# 10711454001)
- **Barcoded ssRNA adaptors** (from Shishkin, et al. (2015), with a blocking group on their 3' ends (*3SpC3)* to avoid self-ligation)[2]:
  - 100 nL droplets containing 0.4 µM of adaptor and 20 mM trehalose were spotted and pre-dried in each well.

    - An example of a barcoded RNA adaptor, barcode sequence in red:
    - 5Phos/**rArUrGrArArUrU***rArGrArUrCrGrGrArArGrArGrCrGrUrCrGrUrG***rU***rArG*/3SpC3/
- **T4 RNA ligase solution** (added to mixing wells with 20 nL carrier wells):
    - 3.1x T4 RNA ligase buffer (NEB, Cat.# MO437M)
    - 3.1 mM ATP (Roche, Cat.# 11140965001)
    - 1 µg/µL BSA (Roche, Cat.# 10711454001)
    - 0.6 U/µL SUPERase-In (Ambion, Cat.#AM2694; 2500U)
    - 5.26 U/µL T4 RNA ligase (NEB, Cat.# MO437M)
    - 25% v/v MyOne Silane magnetic beads (Life Technologies, Cat.# 37002D) washed four times in UltraPure DNase/RNase-free distilled water and mixed with the UltraPure DNase/RNase-free distilled water to restore the original volume.
    - UltraPure DNase/RNase-free distilled water (Invitrogen, Cat.# 10977-015)
- **PEG solution** (added to mixing wells with 28.6 nL carrier wells):
    - 39.5% PEG8000 (Sigma, Cat.# 83271-100ML-F, PCode:101129041)
    - 17.5% v/v MyOne Silane magnetic beads (Life Technologies, Cat.# 37002D) washed four times in UltraPure DNase/RNase-free distilled water and mixed with the UltraPure DNase/RNase-free distilled water to restore the original volume.
    - 0.7 µg/µL BSA (Roche, Cat.# 10711454001)
- **Wash buffer** (added t)**:**
    - 0.05% NP-40 Surfact-Amps™ Detergent Solution (ThermoFisher Scientific, Cat.# 85124)
    - 0.5 U/µL SUPERase-In (Ambion, Cat.# AM2694)
    - 1 µg/µL BSA (Roche, Cat.# 10711454001)
    - All reagents were dissolved in 1x TE buffer, pH 7.5 (Affymetrix, Cat.# PN733893).

Supplementary References

1. Y. Kang, I. McMillan, M. H. Norris and T. T. Hoang, *Nat. Protoc.*, 2015, **10**, 974.
2. A. A. Shishkin, G. Giannoukos, A. Kucukural, D. Ciulla, M. Busby, C. Surka, J. Chen, R. P. Bhattacharyya, R. F. Rudy and M. M. Patel, *Nat. Methods*, 2015, **12**, 323.

Contributions of Non-Corresponding Authors

Dmitriy V. Zhukov

- Based on the initial prototype by D.A.S., improved, fabricated, and tested initial drop-in device prototypes.
- Designed, fabricated, and tested final drop-in device prototypes, with feedback from E.M.K.
- Performed experiments to generate data for figures 3 and 4.
- Performed on-device steps of the experiments to generate data for figure 6, together with E.M.K.
- Analyzed sequencing data results for figure 6.
- Generated figures 1, 2, 3, 4, 5 (right panel), 6, S1, S2, S3, S4.
- Contributed to writing of all sections of the manuscript and supporting information.

Eugenia M. Khorosheva

- Major contributor to the idea of making the device for barcoding for RNAseq.
- Re-designed RNAtag-Seq protocol (from extraction to barcoding) to perform it as an additive protocol on device. Key additions/changes: selected published lysis methods that work for bacteria and does not impair ligation performance; each step works well for small initial number of loaded RNA molecules; used surfactants, and step-wise added buffers allow for performing a pipeline of biochemical reactions on device without any intermediate clean ups; wash buffer stops ligation and allow for pooling nucleic acids well enough so the loss on water/oil interface is neglectable.
- Contributed to writing Experimental and SI sections.

Tahmineh Khazaei

- Developed pipeline to process and analyze sequencing data.
- Analyzed data shown in figure 6.

Wenbin Du

- Theorized the drop-in idea in the context of microfluidic SlipChip devices.
- Generated figure 5 (left panel).

David A. Selck

- Designed the initial drop-in device prototype.
- Optimized the automatic spotting process.

Alexander A. Shishkin

- Major contributor to the idea of making the device for barcoding for RNAseq.
- Re-designed RNAtagSeq protocol off device to work for small initial number of loaded RNA molecules. Key additions/changes: suggested addition UMIs to p38 sequence, suggested modified R14 and P38 sequences; optimized intermediate clean up between off device reactions using MyOneSilane beads and final 0.6 - 0.7 v/v EtOH for size selection.
